# Airway Changes after Cleft Orthognathic Surgery Evaluated by Three-Dimensional Computed Tomography and Overnight Polysomnographic Study

**DOI:** 10.1038/s41598-017-12251-4

**Published:** 2017-09-25

**Authors:** Chun-Shin Chang, Christopher Glenn Wallace, Yen-Chang Hsiao, Yuh-Jia Hsieh, Yi-Chin Wang, Ning-Hung Chen, Yu-Fang Liao, Eric Jen-Wein Liou, Philip Kuo-Ting Chen, Jyh-Ping Chen, Yu-Ray Chen

**Affiliations:** 1 Department of Chemical and Materials Engineering, College of Engineering, Chang Gung University, No. 259, Wenhua 1st Rd., Guishan Dist., Taoyuan City, 33302 Taiwan (R.O.C.); 2 Craniofacial Research Center, Department of Medical Research, Department of Plastic & Reconstructive Surgery, Chang Gung Memorial Hospital, 5, Fu-Hsin St., Guei-Shan 333, Taoyuan, Taiwan (R.O.C.); 3 Craniofacial Research Center, Department of Medical Research, Department of Craniofacial Orthodontics, Chang Gung Memorial Hospital, 5, Fu-Hsin St., Guei-Shan 333, Taoyuan, Taiwan (R.O.C.); 4 Sleep Center, Department of Pulmonary and Critical Care Medicine, Chang Gung Memorial Hospital, 5, Fu-Hsin St., Guei-Shan 333, Taoyuan, Taiwan (R.O.C.)

## Abstract

Cleft lip and palate is the most common congenital craniofacial anomaly. Up to 60% of these patients will benefit from cleft orthognathic surgery, which consists primarily of maxillary advancement and mandibular setback to address midface retrusion and relative mandibular protrusion, respectively. It is believed that maxillary advancement can enlarge the airway whilst mandibular setback can reduce the airway, but this has not previously been quantified for cleft patients undergoing orthognathic surgery. This unique longitudinal prospective study of 18 patients was conducted between April 2013 and July 2016. No significant changes occurred by six months postoperatively in body mass index, apnoea-hypopnoea index or lowest oxygen saturation (LSAT). There was a mean increase of 0.73 cm^3^ in velopharyngeal volume, a mean decrease of 0.79 cm^3^ in oropharyngeal volume, an improvement in snoring index, and no statistically significant change in hypopharyngeal volume. In conclusion, cleft orthognathic surgery that produced anterior advancement of the maxilla, setback of the mandible and clockwise rotation of the maxillo-mandibular complex resulted in increased velopharyngeal, decreased oropharyngeal and unchanged hypopharyngeal airways, and improved snoring, but did not significantly alter objective sleep-related breathing function.

## Introduction

Our Craniofacial Center multidisciplinary team includes craniofacial plastic surgeons, craniofacial orthodontists, speech therapists, social workers and other subspecialists. Since 1976, this team has treated more than 30,000 cleft patients and more than 10,000 of them have received orthognathic surgery.

During more than three decades, our Craniofacial Center has progressively optimised our management strategies for our patients with cleft lip and palate. The primary lip and palate repair carried out during infancy and early childhood lays the foundation for providing an aesthetic facial appearance and speech that is normal. One long-term negative effect of these early surgical interventions is a significant incidence of maxillary growth restriction that produces secondary deformities of the jaws and malocclusion, which affects speech, airway and self-esteem^1^.

Up to 60% of cleft patients will require orthognathic surgery^2^. If a patient has residual maxillofacial deformities (mid-face retrusion and mandibular protrusion) in adolescence, our management is to combine Le Fort I maxillary advancement and bilateral sagittal split setback with single splint techniques^3,4^.

Maxillo-mandibular advancement can enlarge the pharyngeal airway and is therefore used to treat obstructive apnoea^5^. Airway obstruction is frequent in cleft children who undergo speech surgical intervention^6–8^. However, the effects of cleft orthognathic surgery (maxillary advancement and mandibular setback) on the airway are still unknown. Accordingly, the objective of this study is to evaluate airway changes after cleft orthognathic surgery.

## Results

Eighteen patients (9 males; 9 females) completed the study. Mean age at time of operation was 19.72 ± 3.30 years. BMI was 20.36 ± 3.16 before surgery and 20.10 ± 2.92 six months after surgery (*p* = 0.247). No surgical complications (eg. infection, post-operative bleeding, gingival retraction, dental injury, etc) were encountered. Polysomnographic, demographic data and nasal septum deviation (NSD) scores are provided in Table 1. There were no significant changes in AHI/hr (1.99 ± 2.90 vs 1.86 ± 2.69; *p* = 0.81), LSAT (90.89 ± 5.85 vs 92 ± 4.90; *p* = 0.168) or NSD (*p* = 1.000) before and after surgery. The snoring index significantly improved after surgery (78.11 ± 113.83 vs 29.18 ± 46.88; *p* = 0.022).

**Table 1:** Demographic, polysomnographic results and nasal septum deviation scores.

Patient No.	Age	Sex	BMI		AHI/hr		LSAT(%)		NSD scores	
			Pre	Post	Pre	Post	Pre	Post	Pre	Post
1	18	M	20.8	21.0	0.4	0.9	93	93	2	2
2	18	M	24.0	22.1	1.8	0.2	91	94	1	1
3	18	M	17.6	18.1	0	1.4	95	89	1	1
4	21	F	19.1	19.5	0	0.5	95	95	1	1
5	18	F	18.1	16.2	0.5	0	91	94	1	1
6	24	M	22.3	23.1	5.3	0	92	93	1	1
7	18	F	22.3	21.9	6.4	0.5	89	91	1	1
8	16	F	17.3	17.7	0.2	0.2	94	95	1	1
9	18	M	16.5	16.5	0.2	0	94	95	1	1
10	27	M	25.3	25.3	9.7	9.3	72	76	1	1
11	18	M	22.4	23.0	0	2.9	93	89	1	1
12	16	F	20.5	20.4	0.2	0.4	95	94	1	1
13	18	F	19.2	19.2	0	0.4	94	96	1	1
14	18	F	23.4	22.7	0	0	98	97	1	1
15	18	M	17.0	17.3	6.2	6.3	83	91	1	1
16	22	F	14.7	14.9	0.8	0.9	91	97	1	1
17	23	M	25.6	23.4	2.1	4.8	87.	89.	1	1
18	26	F	20.3	19.5	2.1	4.8	89	88.	1	1
Mean	19.72		20.36	20.10	1.99	1.86	90.89	92.00		
Standard Deviation	3.30		3.16	2.92	2.90	2.69	5.85	4.90		
p			0.247		0.810		0.168		1.000	

Fourteen patients reported “Marked improvement” and four patients reported “Some improvement” in their facial appearance with surgery. None reported “No improvement” in, or “Worse”, facial appearance.

Facial skeletal landmark movements are provided in Tables 2 and 3. The A points were advanced by mean 4.41 mm (*p* < 0.001) and moved inferiorly by mean 3.33 mm (*p* < 0.001). The posterior nasal spines (PNSs) were advanced by mean 5.22 mm (*p* < 0.001); the PNSs were not significantly moved in the vertical dimension (*p* = 0.331). The B points, pogonions (POGs) and genioglossal tubercles (GGTs) were respectively moved posteriorly by means of 4.08 mm, 4.79 mm and 4.84 mm (*p* = 0.002, *p* = 0.002 and *p* = 0.001), and were moved inferiorly by means of 2.47 mm, 3.76 mm and 3.41 mm (*p* = 0.001, *p* = 0.002 and *p* = 0.001). The lower central incisor points (LCIs; defined as the most superior point on the junction between the two lower central incisors) were moved posteriorly by mean 4.28 mm (*p* < 0.001) and inferiorly by mean 2.16 mm (*p* = 0.009). The occlusal plane altered significantly by a mean of 6.61 degrees (*p* < 0.001) (Table 4).

**Table 2:** Movement of facial landmarks; Horizontal (data presented in mm, mean ± standard deviation).

Landmark	Before surgery	6-months after surgery	Mean difference	Paired-T test *p* value
A	52.62 ± 4.12	57.02 ± 4.39	−4.41 ± 2.41	<0.001
PNS	19.24 ± 4.69	24.47 ± 4.50	−5.22 ± 2.16	<0.001
B	53.12 ± 6.47	49.04 ± 5.68	4.08 ± 4.80	0.002
Pog	52.74 ± 8.14	47.95 ± 7.30	4.79 ± 5.60	0.002
GGT	38.18 **±** 7.56	33.34 ± 6.48	4.84 ± 4.89	0.001
LCI	61.92 ± 5.30	57.64 ± 6.09	4.28 ± 3.95	<0.001

**Table 3:** Movement of facial landmarks; Vertical (data presented in mm, mean ± standard deviation).

Landmark	Before surgery	6-months after surgery	Mean difference	Paired-T test *p* value
A	52.93 ± 4.17	56.26 ± 4.94	−3.33 ± 2.44	<0.001
PNS	46.67 ± 4.30	46.19 ± 4.81	0.47 ± 1.98	0.331
B	88.77 ± 6.76	91.24 ± 7.48	−2.47 ± 2.60	0.001
Pog	105.96 ± 9.22	109.72 ± 9.47	−3.76 ± 4.35	0.002
GGT	102.24 ± 8.69	105.64 ± 10.09	−3.41 ± 3.71	0.001
LCI	72.26 ± 5.11	74.42 ± 7.42	−2.16 ± 3.11	0.009

**Table 4:** Occlusal plane.

Landmark	Before surgery	6-months after surgery	Mean difference	Paired-T test *p* value
Occlusal plane Angle	7.67 ± 2.58	14.28 ± 3.82	−6.61 ± 3.53	<0.001

Changes in airway dimensions are provided in Tables 5, 6 and 7. There were no statistically significant changes in the minimum LSAT measurements at velopharyngeal, oropharyngeal and hypopharyngeal airway levels. There was a mean increase of 0.22 cm in the minimum AP measurement at the velopharyngeal level (*p* = 0.005), a mean decrease of 0.15 cm at the oropharyngeal level (*p* = 0.007), and no statistically significant change at the hypopharyngeal level. There was a mean increase of 0.46 cm^2^ in the minimum CSA at the velopharyngeal level (*p* < 0.001), a mean decrease of 0.81 cm^2^ at the oropharyngeal level (*p* = 0.025), and no statistically significant change at the hypopharyngeal level. There was a mean increase of 0.73 cm^3^ in velopharyngeal volume (*p* = 0.028), a mean decrease of 0.79 cm^3^ in oropharyngeal volume (*p* = 0.038), and no statistically significant change in hypopharyngeal volume and total pharyngeal volume.

**Table 5:** Changes in the pharyngeal airway; Minimum linear lateral and anterior-posterior measurements at three pharyngeal airway segments.

LAT & AP (cm)	Before surgery	6-months after surgery	Mean difference	Paired-T test *p* value
LAT-VP	2.35 ± 0.78	2.17 ± 0.65	0.18 ± 0.46	0.110
LAT-OP	2.61 ± 0.91	2.41 ± 0.65	0.20 ± 0.57	0.150
LAT-HP	3.19 ± 0.55	3.07 ± 0.52	0.11 ± 0.44	0.292
AP-VP	1.20 ± 0.33	1.43 ± 0.46	−0.22 ± 0.30	0.005
AP-OP	1.28 ± 0.44	1.12 ± 0.37	0.15 ± 0.22	0.007
AP-HP	1.45 ± 0.55	1.37 ± 0.39	0.07 ± 0.38	0.429

**Table 6:** Changes in the pharyngeal airway; Minimum area measurements at three pharyngeal airway segments.

Minimum Area (cm^2^)	Before surgery	6-months after surgery	Mean difference	Paired-T test *p* value
VP	2.67 ± 1.88	3.13 ± 1.86	−0.46 ± 0.42	<0.001
OP	2.63 ± 1.93	1.82 ± 1.56	0.81 ± 1.40	0.025
HP	3.20 ± 1.86	2.79 ± 1.53	0.41 ± 1.08	0.125

**Table 7:** Changes in the pharyngeal airway; Volume measurements at three pharyngeal airway segments and total pharyngeal airway.

Volumes (cm^3^)	Before surgery	6-months after surgery	Mean difference	Paired-T test *p* value
VP	5.67 ± 3.18	6.40 ± 3.28	−0.73 ± 1.29	0.028
OP	4.45 ± 2.58	3.65 ± 2.15	0.79 ± 1.51	0.038
HP	7.20 ± 3.85	7.33 ± 3.33	−0.13 ± 1.81	0.759
Total	17.31 ± 8.39	17.38 ± 7.53	−0.07 ± 2.69	0.915

## Discussion

Cleft lip and palate is the most common congenital facial anomaly. There is a need for multidisciplinary care for these patients during many stages of their lives^9–11^. This study is typical of complete cleft care, involving multidisciplinary treatment and analyses of their treatment outcomes.

When cleft lip/palate patients grow, maxillary retrusion is frequently noted. The main cause of reduced anterior-posterior maxillary growth is the effect of scar tissue formation from surgical cleft lip repair, gingivoperiosteoplasty, hard palate closure and alveolar bone grafting^12–18^. It has been estimated that up to 60% of cleft patients require Le Fort I osteotomies to correct maxillary hypoplasia^2^.

Growth restriction causes the maxilla to become deficient and retrusive, leading to a prognathic facial appearance^19^. The patients in this current study all presented some degree of mandibular prognathism, demonstrated by increased SNB (Table 1). Such discrepancies in maxillary and mandibular development can cause severe class III malocclusion. The surgical correction of maxillary hypoplasia and cleft malocclusion with conventional orthognathic surgery (Le Fort I and bilateral sagittal split osteotomy) is generally conducted after skeletal maturity. Adjunctive orthodontic treatments are essential. Computer-aided 3D surgical planning using CT scans is performed by the orthodontic team in our institution and has improved surgical outcomes^20,21^. Orthognathic surgery for cleft patients is intended to improve dental occlusion, skeletal relationships and facial appearance^22^. Of the 18 patients studied, 14 (78%) reported a marked improvement and four (22%) reported some improvement in their facial appearance with orthognathic surgery.

**Figure 1:**
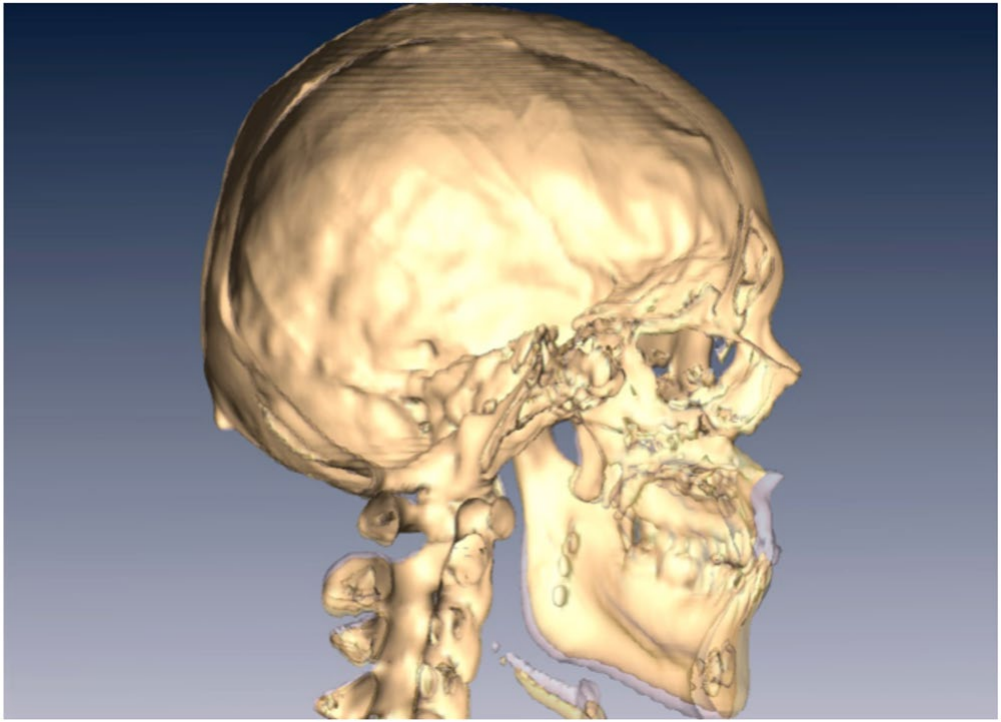
Superimposition of three dimensional CT scans before (yellow) and six months after surgery (light purple). Notice the maxilla has been advanced and the mandible has been moved posteriorly. There is also clockwise rotation of the entire maxilla-mandible complex.

To correct a hypoplastic maxilla with inadequate incisor show, typically the maxilla is advanced and moved inferiorly in the anterior portion (demonstrated by movement of the A point), and rotated clockwise at the PNS relative to its vertical position. The mandibular body is conversely moved posteriorly. Overall, the maxilla-mandibular complex is clockwise rotated and the mandibular body moved inferiorly. This is demonstrated as setback and inferior movement of the B points, POGs, GGTs and LCIs (Tables 2 and 3; Fig. 1).

**Figure 2:**
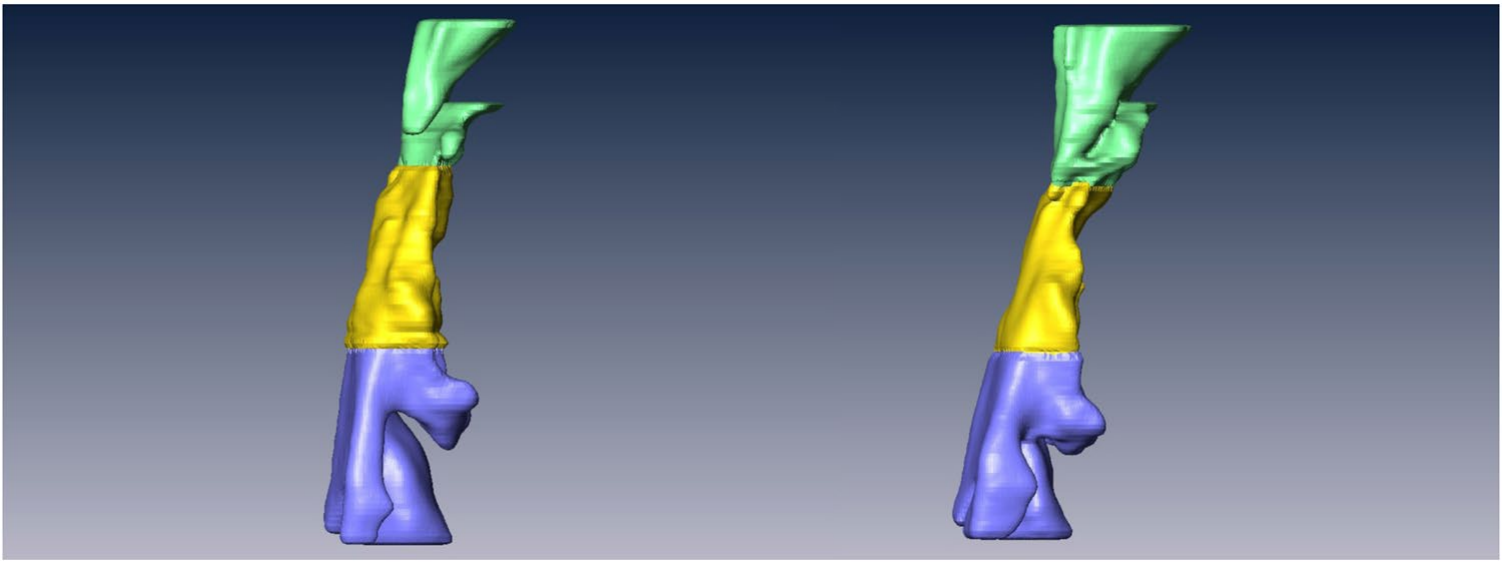
Three-dimensional airway model before (left) and after (right) surgery. Notice that the velopharyngeal airway has increased, the oropharyngeal airway has decreased, and the hypopharyngeal airway has not changed.

**Figure 3:**
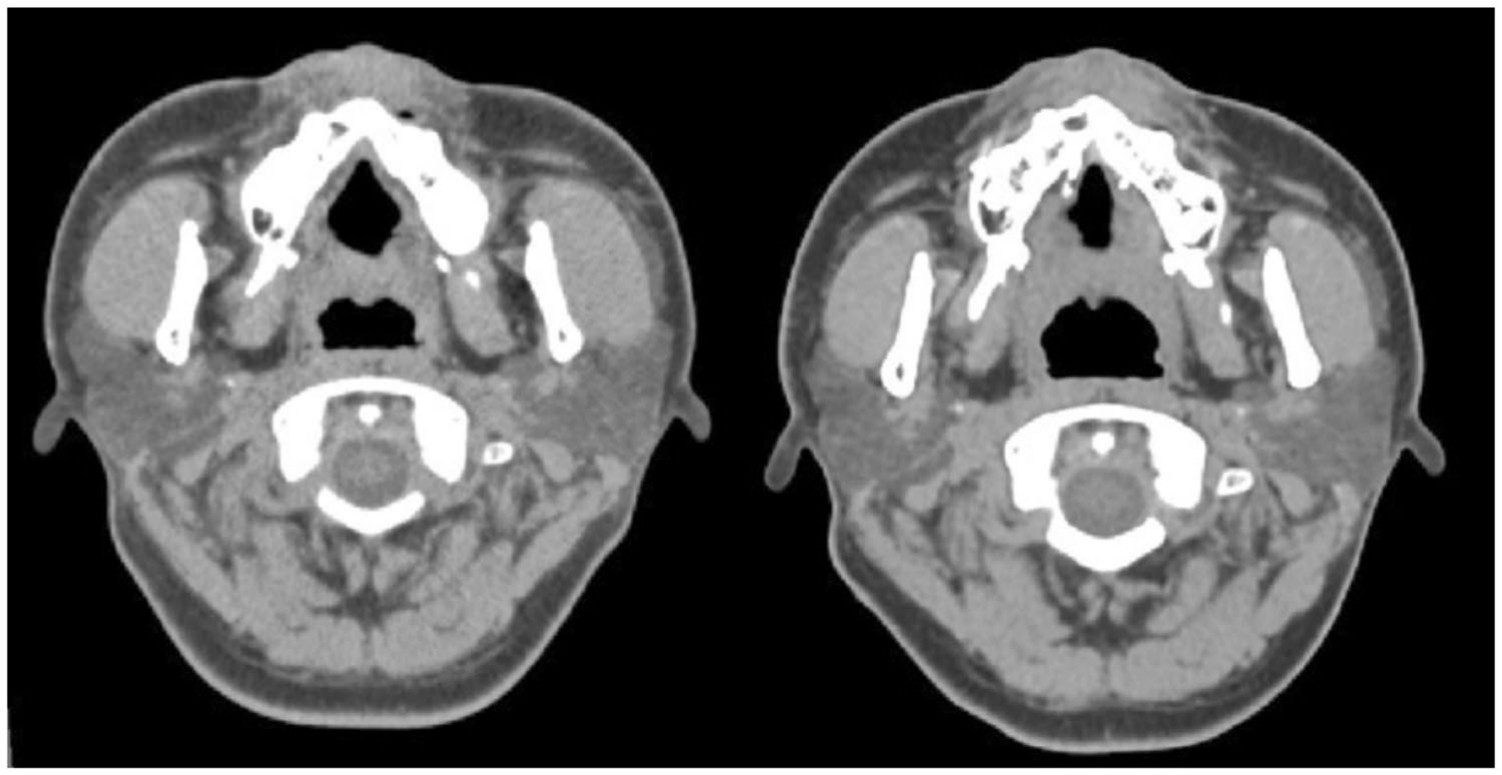
Minimum area measurement of the velopharyngeal airway (left is before surgery; right is after surgery). Notice the increase in AP distance.

**Figure 4:**
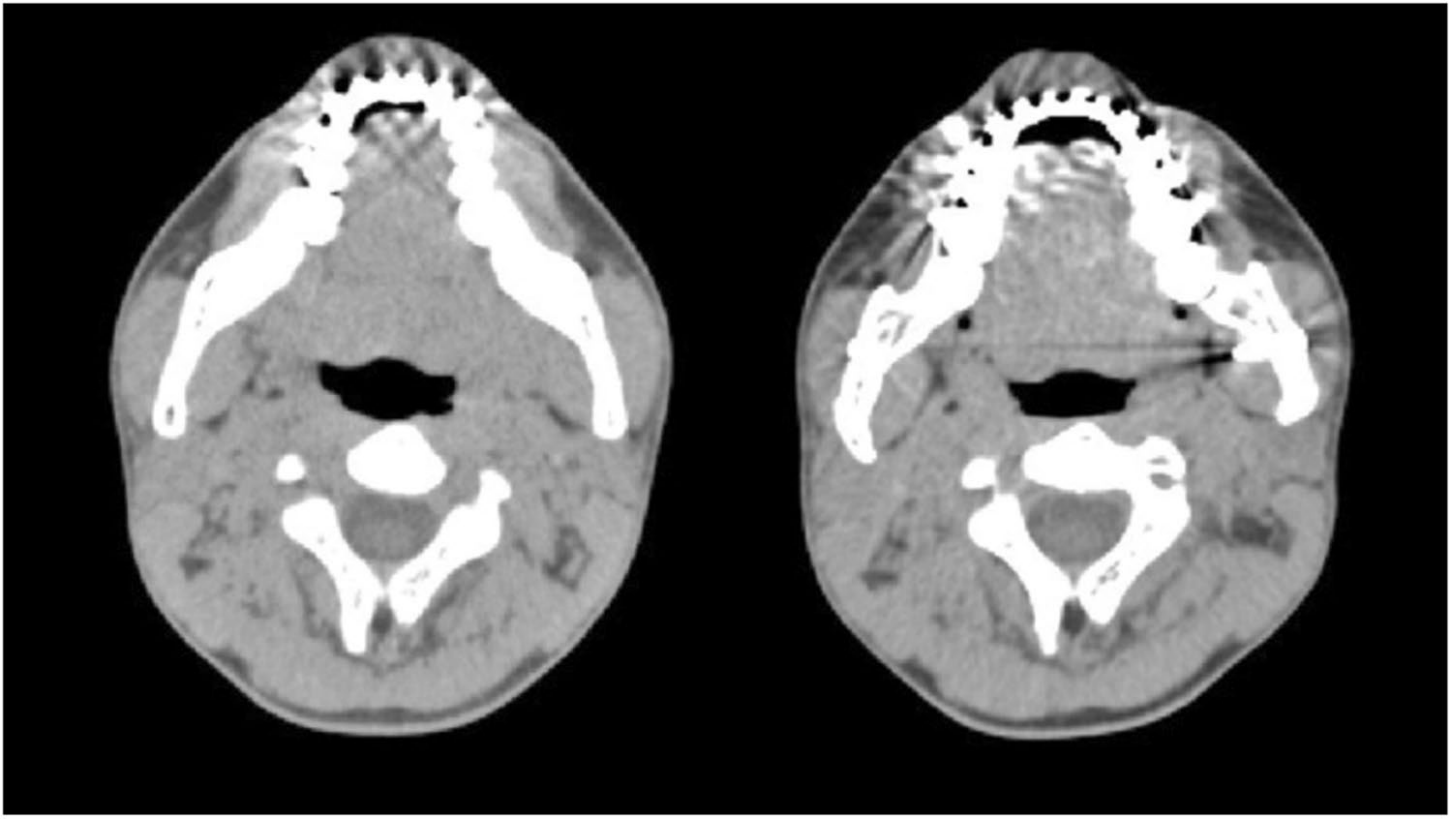
Minimum area measurement of the oropharyngeal airway (left is before surgery; right is after surgery). Notice the decrease in AP distance.

**Figure 5:**
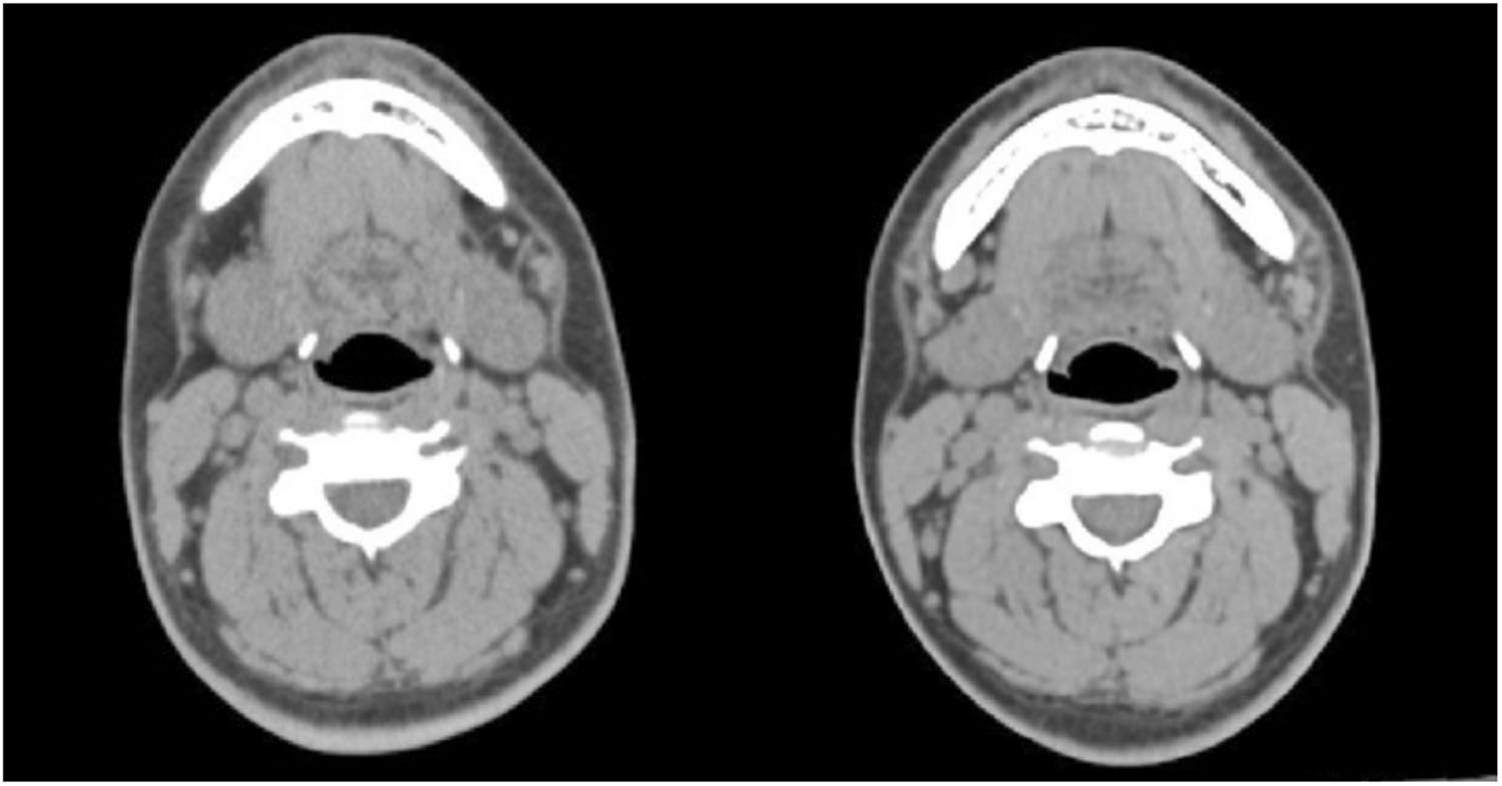
Minimum area measurement of the hypopharyngeal airway (left is before surgery; right is after surgery). Notice the lack of change in AP and LAT distances.

The 2-D and 3-D influences on the airway of maxillary advancement and mandibular setback plus clockwise rotation of the maxilla-mandibular complex in cleft orthognathic surgery have never been reported in the literature. Mandibular setback surgery for skeletal class III malocclusion or mandibular prognathism caused decreases in the airway dimensions^23,24^, and maxillary advancement produced increased upper airway dimensions^25,26^. However, cleft orthognathic surgery requires both maxillary advancement and mandibular setback. The present study demonstrated a postsurgical increase in velopharyngeal volume from 5.67 cm^3^ to 6.40 cm^3^, a decrease in oropharyngeal volume from 4.45 cm^3^ to 3.65 cm^3^, and no significant changes in hypopharyngeal and total pharyngeal volumes (Fig. 2). These three-dimensional changes were also observed in the minimum cross-sectional area measurements at the three levels of the airway. There was an increase of minimum cross-sectional area at the velopharyngeal airway (Fig. 3), a decrease at the oropharyngeal airway (Fig. 4) and no significant change at the hypopharyngeal airway (Fig. 5). These airway changes were mainly due to AP distance rather than LAT distance changes (Figs 1, 2, 3, 4 and 5). However, these airway changes, as measured by 3D CT scans, did not translate into objective respiratory functional changes during sleep.

Although cleft lip-palate patients frequently complain of snoring and respiratory difficulties during sleep, overnight polysomnographic studies are not commonly performed as a presurgical assessment. Our findings suggest that cleft orthognathic surgery did not significantly alter these patients’ AHIs or LSAT readings six months after surgery.

### Strengths and limitations of this study

This study has several strengths. First, our Craniofacial Center is a major teaching hospital referral centre that has treated most of the cleft patients in our nation. All patients were consecutive consenting patients from the first and corresponding authors’ cleft practices and both surgeons exercise the same surgical approach to cleft orthognathic surgery; thus, selection bias is minimised. Second, the study group has extensive experience in airway measurement techniques, and have already published several peer-review articles regarding airway measurement using 3 dimensional CT scan technology^5,23,25^. Third, given that Taiwan is a small island, complete six months follow up for most of our patients was achieved.

The limitations of this study merit emphasis. First, although the Craniofacial Center treats several hundred patients each year with orthognathic surgery, most patients declined to be included in the present investigation due to the need for two overnight polysomnographic studies. Second, we included only unilateral complete cleft lip and palate patients, but patients with bilateral complete cleft lip and palate tend to have more severe midface retrusion and would warrant further study. Third, in our institution more than 90% of patients proceeding with cleft orthognathic surgery have bimaxillary surgery for aesthetics. Airway and polysomnographic changes in patients electing only for maxillary advancement invites further study. Fourth, most of our patients did not have, or only had mild, obstructive sleep apneoa before surgery. Further study in cleft patients with moderate to severe obstructive sleep apneoa warrants future investigation.

We consider patients with objectively small airway, obesity, and/or symptoms, signs or family history of obstructive sleep apnea, as high risk for airway compromise following mandibular setback; their presence may lead us to discourage mandibular setback. Other unfavourable characteristics for airway risk following mandibular setback appear to be male sex and middle agedness. Careful surgical planning is important so as not to compromise basic airway needs after mandibular setback^23^.

In conclusion, cleft orthognathic surgery that produced anterior advancement of the maxilla, setback of the mandible and clockwise rotation of the maxillo-mandibular complex resulted in increased velopharyngeal, decreased oropharyngeal and unchanged hypopharyngeal airways, and improved snoring, but did not significantly alter objective sleep-related breathing function.

## Methods

This prospective longitudinal study was designed to investigate skeletal changes, two and three dimensional airway changes, polysomnographic changes and patient reported satisfaction with facial appearance after cleft orthognathic surgery.

### Ethics

This study was approved by the Institutional Review Board (IRB) of Chang Gung Memorial Hospital (IRB #101–5046A3) on the 12^th^ April 2013. All methods were performed in accordance with the relevant guidelines and regulations. Patient recruitment commenced on the 22^nd^ June 2013 and the final patient was recruited on the 28^th^ November 2015. Follow-up for the final patient completed on the 22^nd^ July 2016. All patients provided IRB-approved fully informed written consent for inclusion into this study.

Data sharing statement, the data can be assessed: https://www1.cgmh.org.tw/intr/intr2/c32540/en/clinical_trials_studies.html; extra data is available by emailing plastreconst@gmail.com.

Twenty-two complete unilateral cleft lip patients with class III malocclusion were treated with orthognathic surgery at the Craniofacial Center of Chang Gung Memorial Hospital from 1992–1996, and were included in the present study. Eighteen patients completed the study; all underwent overnight polysomnography and computed tomography (CT) scans before and at 6 months after orthognathic surgery. Demographic data was recorded (age, sex, body mass index; BMI). The BMI was recorded at the time of polysomnography, before surgery and at 6 months after surgery.

The inclusion criteria were: 1. unilateral cleft lip/palate patients; 2. patients with midface retrusion and malocclusion that required orthognathic surgery; 3. such patients who provided consent for inclusion; 4. patients who had reached skeletal maturation.

Exclusion criteria were: 1. presence of other craniofacial anomalies; 2. soft tissue airway surgery within one year; 3. presence of medical conditions that would preclude orthognathic surgery.

### Orthognathic Surgery

Bimaxillary osteotomies were executed according to three-dimensionally planned surgical movements^21^. All patients underwent single splint orthognathic surgery^3^. All patients received combined Le Fort I maxillary osteotomies to advance the maxilla and bilateral sagittal split ramus osteotomies to set back the mandible. All surgical complications were recorded.

### Measurements

All measurements and data analyses were performed by a single investigator. CT scan data was stored in digital imaging and communications in medicine (DICOM) format and was transferred to a computer station with AVIZO version 7.01 software (VSG, France). The 3-dimensional CT images were first oriented as follows: axial plane was the Frankfort Horizontal (FH) plane (defined by the plane passing through bilateral orbitale and bilateral porion); coronal plane was perpendicular to the FH plan; sagittal plane was perpendicular to the FH and coronal planes, this passed through the midpoint of the bilateral orbitale. Three dimensional CTs (before and six months after surgery) were superimposed in cranial structures before measurements.

**Figure 6:**
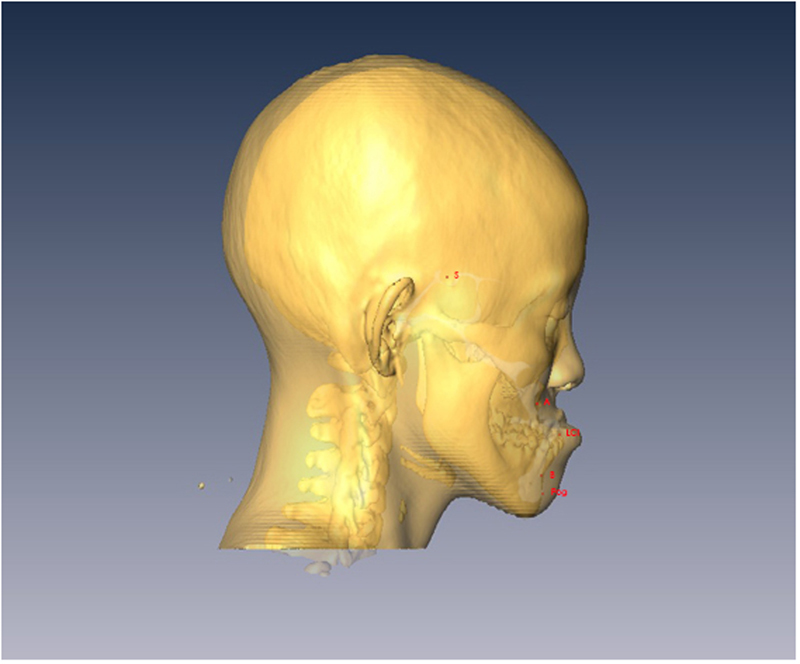
Facial skeletal movements before and after surgery were measured in vertical and horizontal distances from the landmarks to the sella. The A point is the most posterior mid-sagittal point on the anterior maxillary surface. The B point is the most posterior mid-sagittal point on anterior mandibular surface. The lower central incisor point (LCI) is the most superior point on the junction between the two lower central incisors. The pogonion (Pog) is the most anterior mid-sagittal point of the chin. The genioglossal tubercle (GGT) is located at the posterior surface of the body of the mandible.

**Figure 7:**
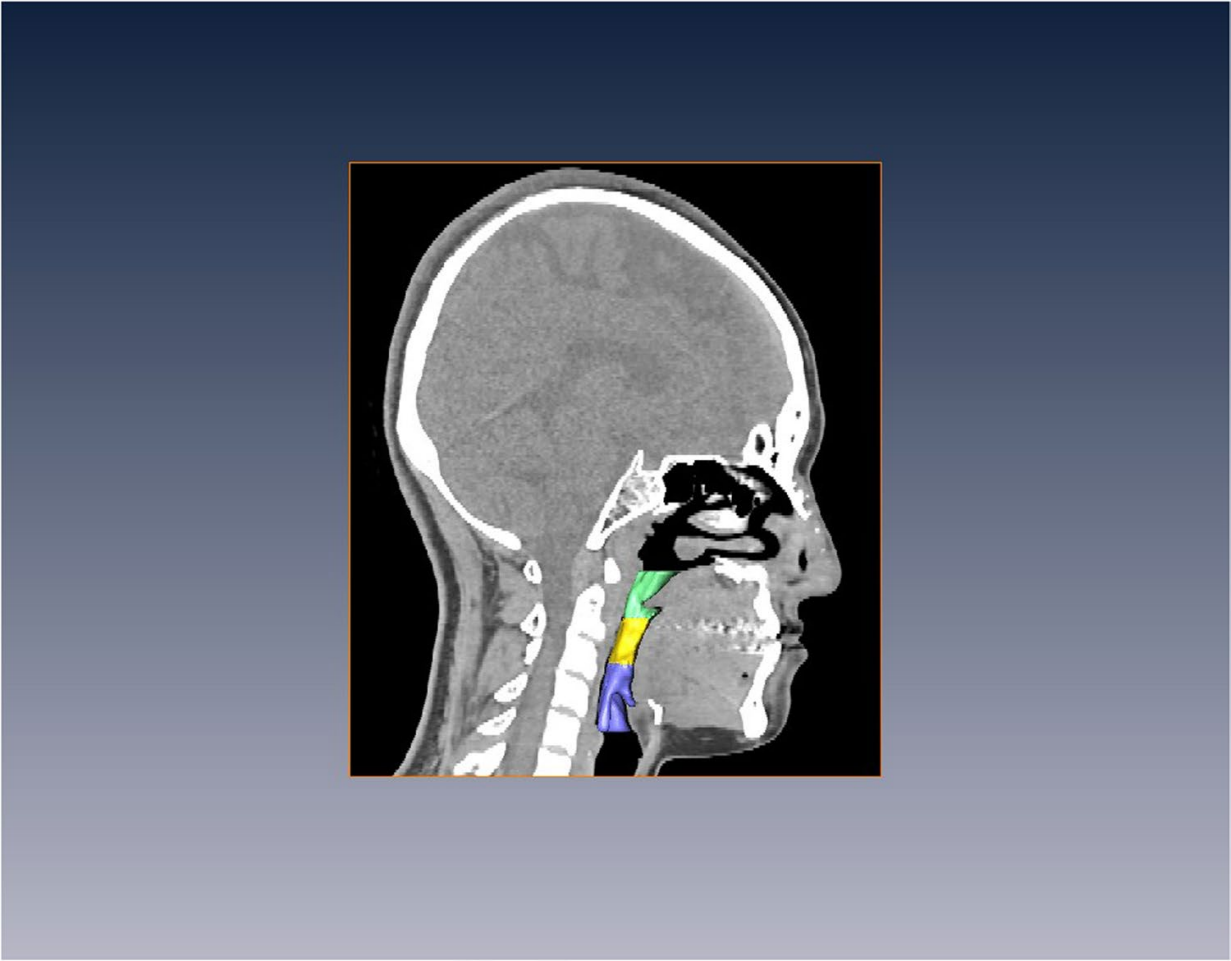
Pharyngeal airway volume: (**A**) Velopharyngeal airway (VP) with the upper margin of the velopharynx as a horizontal plane that is perpendicular to the sagittal plane and passes through the posterior nasal spine; and the lower margin of the velopharynx as a horizontal plane that is perpendicular to the sagittal plane and passes through the tip of the uvula; (**B**) Oropharyngeal airway with its upper margin as the tip of the uvula and its lower margin as a horizontal plane perpendicular to the sagittal plane that passes through the tip of the epiglottis; (**C**) Hypopharyngeal airway with its upper margin at the tip of the epiglottis and its lower margin as a horizontal plane that is perpendicular to the sagittal plane that passes through the vocal cord.

**Figure 8:**
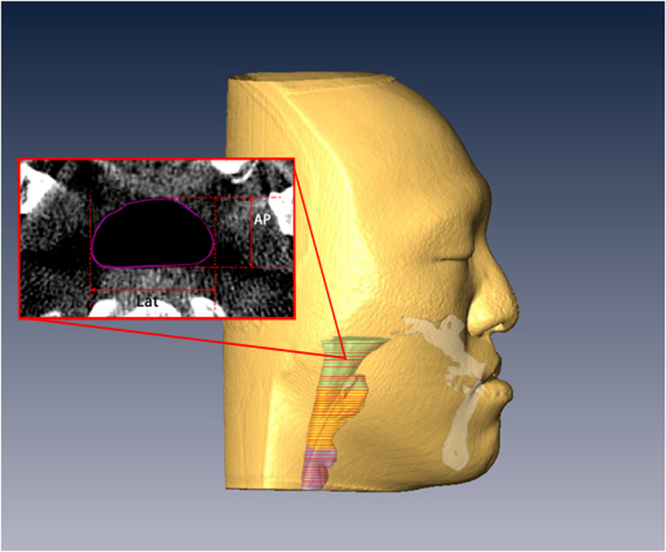
Airway measurements: the anterior-posterior (AP) and lateral (Lat) dimensions, and the cross-sectional area (CSA; in purple) were measured.

Facial skeletal movements before and after surgery were measured using vertical and horizontal distances from the landmarks to the sella (Fig. 6). The occlusal plane angle was that angle between the occlusal plane and the S-N plane. Airway changes, before and after surgery, were measured using 3-dimensional airway modelling as follows. The upper airway was divided into 3 segments: velopharynx, oropharynx and hypopharynx (Fig. 7). The minimum anterior-posterior dimension (AP), lateral dimension (LAT), and cross-sectional area (CSA) were measured at each of these three segments (velopharynx, oropharynx and hypopharynx; Fig. 8). The CT slices with the minimum available measured AP distances for each respective airway level were defined as the CT slices for preoperative and postoperative measurements.

Pre-operative and 6 months post-operative polysomnographic studies and nasal septal deviation (NSD) scores (Grade 1: <33%, Grade 2: 33–66% and Grade 3: >66% deflection from the midline toward the lateral wall^27^) were recorded. The apnoea-hypopnoea index (AHI) and lowest oxygen saturation (LSAT) were obtained at both time points.

Each patient’s reported satisfaction regarding their facial appearance was obtained 6 months after surgery and was graded as (1) Marked improvement; (2) Some improvement; (3) No improvement and (4) Worse.

### Statistical analyses

The paired *t*-test was used to compare facial skeletal movement, upper airway measurements and polysomnographic data. Fisher’s exact test was used to compare NSD scores. All statistical analyses were performed with SPSS 17.0 (SPSS, Inc., Chicago, Ill). A *p* value of less than 0.05 denoted a statistically significant difference. Mean results are provided with standard deviations (mean ± standard deviations).

## References

[1] Posnick JC, Ricalde P (2004). Cleft-orthognathic surgery. Clinics in plastic surgery.

[2] Hathaway R (2011). The Americleft study: an inter-center study of treatment outcomes for patients with unilateral cleft lip and palate part 2. Dental arch relationships. Cleft Palate Craniofac J.

[3] Yu CC, Bergeron L, Lin CH, Chu YM, Chen YR (2009). Single-splint technique in orthognathic surgery: intraoperative checkpoints to control facial symmetry. Plastic and reconstructive surgery.

[4] Bergeron L, Yu CC, Chen YR (2008). Single-splint technique for correction of severe facial asymmetry: correlation between intraoperative maxillomandibular complex roll and restoration of mouth symmetry. Plastic and reconstructive surgery.

[5] Lin CH, Liao YF, Chen NH, Lo LJ, Chen YR (2011). Three-dimensional computed tomography in obstructive sleep apneics treated by maxillomandibular advancement. The Laryngoscope.

[6] Liao YF (2002). Incidence and severity of obstructive sleep apnea following pharyngeal flap surgery in patients with cleft palate. Cleft Palate Craniofac J.

[7] Liao Y-F (2004). Comparison of Obstructive Sleep Apnea Syndrome in Children With Cleft Palate Following Furlow Palatoplasty or Pharyngeal Flap for Velopharyngeal Insufficiency. The Cleft Palate-Craniofacial Journal.

[8] Liao Y-F (2003). Longitudinal Follow-Up of Obstructive Sleep Apnea Following Furlow Palatoplasty in Children With Cleft Palate: A Preliminary Report. The Cleft Palate-Craniofacial Journal.

[9] Cher Bing C (2008). The Continuing Multidisciplinary Needs of Adult Patients With Cleft Lip and/or Palate. The Cleft Palate-Craniofacial Journal.

[10] Taub PJ, Lampert JA (2011). Pediatric Craniofacial Surgery: A Review for the Multidisciplinary Team. The Cleft Palate-Craniofacial Journal.

[11] Han HH, Choi EJ, Kim JM, Shin JC, Rhie JW (2016). The Importance of Multidisciplinary Management during Prenatal Care for Cleft Lip and Palate. Archives of plastic surgery.

[12] Ross RB (1987). Treatment variables affecting facial growth in complete unilateral cleft lip and palate. The Cleft palate journal.

[13] Mars M, Houston WJ (1990). A preliminary study of facial growth and morphology in unoperated male unilateral cleft lip and palate subjects over 13 years of age. The Cleft palate journal.

[14] Liao YF, Mars M (2005). Long-term effects of clefts on craniofacial morphology in patients with unilateral cleft lip and palate. Cleft Palate Craniofac J.

[15] Liao YF, Mars M (2005). Long-term effects of palate repair on craniofacial morphology in patients with unilateral cleft lip and palate. Cleft Palate Craniofac J.

[16] Liao YF, Mars M (2005). Long-term effects of lip repair on dentofacial morphology in patients with unilateral cleft lip and palate. Cleft Palate Craniofac J.

[17] Liao YF, Mars M (2006). Hard palate repair timing and facial growth in cleft lip and palate: a systematic review. Cleft Palate Craniofac J.

[18] Hsieh CH, Ko EW, Chen PK, Huang CS (2010). The effect of gingivoperiosteoplasty on facial growth in patients with complete unilateral cleft lip and palate. Cleft Palate Craniofac J.

[19] Phillips JH, Nish I, Daskalogiannakis J (2012). Orthognathic surgery in cleft patients. Plastic and reconstructive surgery.

[20] Haas OL, Becker OE, de Oliveira RB (2015). Computer-aided planning in orthognathic surgery—systematic review. International journal of oral and maxillofacial surgery.

[21] Lonic D (2016). Computer-Assisted Orthognathic Surgery for Patients with Cleft Lip/Palate: From Traditional Planning to Three-Dimensional Surgical Simulation. PloS one.

[22] Yun YS (2015). Bone and Soft Tissue Changes after Two-Jaw Surgery in Cleft Patients. Archives of plastic surgery.

[23] Hsieh Y-JDDSMS, Chen Y-CMD, Chen Y-AMD, Liao Y-FDDSPD, Chen Y-RMD (2015). Effect of Bimaxillary Rotational Setback Surgery on Upper Airway Structure in Skeletal Class III Deformities. Plastic & Reconstructive Surgery.

[24] Riley RW, Powell NB, Guilleminault C, Ware W (1987). Obstructive sleep apnea syndrome following surgery for mandibular prognathism. Journal of oral and maxillofacial surgery: official journal of the American Association of Oral and Maxillofacial Surgeons.

[25] Hsieh YJ, Liao YF, Chen NH, Chen YR (2014). Changes in the calibre of the upper airway and the surrounding structures after maxillomandibular advancement for obstructive sleep apnoea. The British journal of oral & maxillofacial surgery.

[26] Aksu M (2012). Pharyngeal airway changes associated with maxillary distraction osteogenesis in adult cleft lip and palate patients. Journal of oral and maxillofacial surgery: official journal of the American Association of Oral and Maxillofacial Surgeons.

[27] Salihoglu M, Cekin E, Altundag A, Cesmeci E (2014). Examination versus subjective nasal obstruction in the evaluation of the nasal septal deviation. Rhinology.

